# Live Cell Monitoring of hiPSC Generation and Differentiation Using Differential Expression of Endogenous microRNAs

**DOI:** 10.1371/journal.pone.0011834

**Published:** 2010-07-28

**Authors:** Masakazu Kamata, Min Liang, Shirley Liu, Yoshiko Nagaoka, Irvin S. Y. Chen

**Affiliations:** 1 Department of Microbiology, Immunology and Molecular Genetics, David Geffen School of Medicine, University of California at Los Angeles, Los Angeles, California, United States of America; 2 Department of Molecular and Medical Pharmacology, David Geffen School of Medicine, University of California at Los Angeles, Los Angeles, California, United States of America; Brigham and Women's Hospital, United States of America

## Abstract

Human induced pluripotent stem cells (hiPSCs) provide new possibilities for regenerative therapies. In order for this potential to be achieved, it is critical to efficiently monitor the differentiation of these hiPSCs into specific lineages. Here, we describe a lentiviral reporter vector sensitive to specific microRNAs (miRNA) to show that a single vector bearing multiple miRNA target sequences conjugated to different reporters can be used to monitor hiPSC formation and subsequent differentiation from human fetal fibroblasts (HFFs). The reporter vector encodes EGFP conjugated to the targets of human embryonic stem cell (hESC) specific miRNAs (*miR-302a* and *miR-302d*) and mCherry conjugated to the targets of differentiated cells specific miRNAs (*miR-142-3p*, *miR-155*, and *miR-223*). The vector was used to track reprogramming of HFF to iPSC. HFFs co-transduced with this reporter vector and vectors encoding 4 reprogramming factors (*OCT4, SOX2, KLF4* and *cMYC*) were mostly positive for EGFP (67%) at an early stage of hiPSC formation. EGFP expression gradually disappeared and mCherry expression increased indicating less miRNAs specific to differentiated cells and expression of miRNAs specific to hESCs. Upon differentiation of the hiPSC into embryoid bodies, a large fraction of these hiPSCs regained EGFP expression and some of those cells became single positive for EGFP. Further differentiation into neural lineages showed distinct structures demarcated by either EGFP or mCherry expression. These findings demonstrate that a miRNA dependent reporter vector can be a useful tool to monitor living cells during reprogramming of hiPSC and subsequent differentiation to lineage specific cells.

## Introduction

Human embryonic stem cells (hESCs) have significant therapeutic potential for various diseases, but the generation of these cells from individual patients raises ethical concerns. Recently, a technological breakthrough where somatic cells from mouse and human can be reprogrammed into hESC-like pluripotent cells, termed induced pluripotent stem cells (iPSCs), was made possible through ectopic expression of combinations of reprogramming factors including *OCT4, SOX2, KLF4, cMYC, LIN28* and *NANOG* [1], [2], [3], [4], [5], [6]. Like hESCs, hiPSCs can be self-renewed and have been proven to differentiate into a variety of cell types. Furthermore, iPSCs generated from patient-derived cells can serve as useful tools for potential therapies, drug screening, or to study pathogenesis outside of patients [7], [8], [9]. In order for this potential to be achieved, it is necessary to efficiently monitor the differentiation of these iPSCs into specific lineages.

MicroRNAs (miRNAs) are small non-coding RNAs which regulate gene expression post-transcriptionally (see recent review [10], [11], [12]). miRNAs can be expressed differentially during development and in a tissue-specific fashion [13], [14]. Since the target sequences for miRNAs are small (21 to 25 in size) [15] and act in a relatively context-independent fashion, they can readily be incorporated into vectors together with reporter genes, resulting in reporter expression that is downregulated only in the presence of the endogenous miRNA within cells.

Previous studies demonstrated the utility of such miRNA regulated reporter vectors to distinguish between somatic cells in distinct differentiation lineages and throughout the course of differentiation [13], [16], [17]. The miRNA target sequence for *miR-142-3p* was inserted after the transgene and expressed in the same mRNA transcript in the context of a lentiviral vector. This vector expression was specifically suppressed in hematopoietic lineages and successfully used to eliminate off-target expression of transgenes [13]. They further showed the effectiveness of regulation of transgene expression by cell type dependent miRNA expression using hESC in pre- and post-differentiated conditions [18].

Here, we use a similar reporter vector sensitive to differentiation specific miRNAs to show that a single vector bearing multiple miRNA target sequences conjugated to different reporters can be used to monitor hiPSC formation from human fibroblasts and subsequent differentiation of the hiPSC.

## Results

### Characterization of a miRNA dependent reporter vector that distinguishes pluripotent cells from differentiated progeny

We constructed a bidirectional vector whereby the reporter gene mCherry is conjugated with perfectly complementary miRNA target sites for *miR-223*, *miR-155*, *miR-142-3p* and EGFP expressed in the anti-sense direction conjugated with *miR-302a* and *miR-302d* (*miR-302 a/d*). The *miR-302* gene encodes a cluster of eight miRNAs on chromosome 4 (miR-302b*-b-c*-c-a*-a-d-367) that are preferentially expressed in embryonal carcinoma cells, hESCs and hiPSCs [19], [20], [21]. Whereas *miR-223*, *miR-155*, and *miR-142-3p* are enriched in differentiated cells [14], [22], *miR-223* and *miR-142-3p* are found in cells of primarily hematopoietic origin [23]. *miR-155* is found in hematopoietic cells as well as in many types of lymphoma and solid cancers [24], [25], [26], [27]. *miR-155* is also expressed 20–50 fold higher in fibroblasts than in hESCs and hiPSCs [21]. Thus, endogenous expression of the miRNAs segregated by differentiation state would result in ablation of EGFP, but not mCherry in pluripotent stem cells and, conversely, ablation of mCherry, but not EGFP in differentiated cells of hematopoietic or fibroblast lineage, or in various malignant cells.

**Figure 1:**
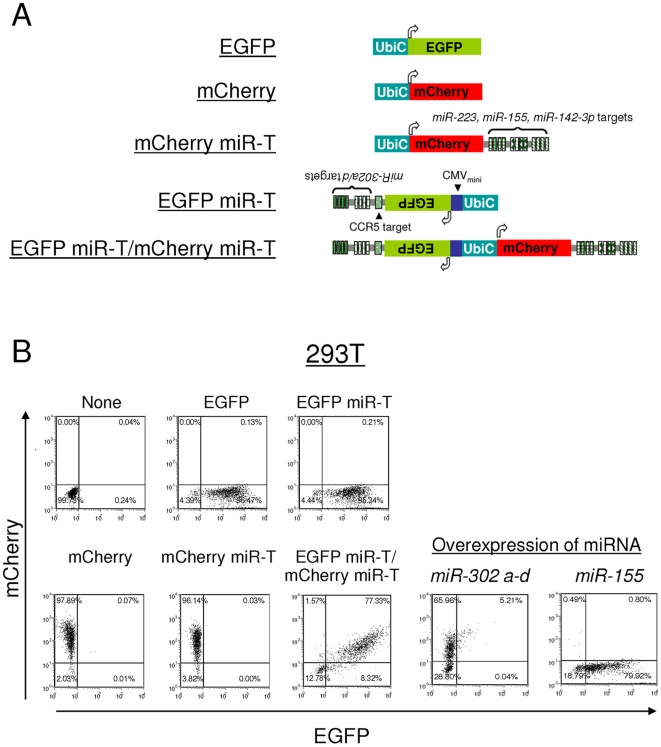
Ectopic expression of the miRNAs specifically suppresses expression of the reporter vector containing miRNA targets in 293T cells. (A) Map of transcriptional units of the reporter vector used in this study. CMVmini: CMV minimal promoter. UbiC: ubiquitin C promoter. CCR5 target: siRNA against CCR5 target sequence (5′-GAGCAAGCTCAGTTTACACC-3′) [59]. (B) 293T cells were infected with lentiviral vectors encoding various reporters shown in (A). Expression levels of EGFP and mCherry were analyzed by flow cytometry 2 days post-infection. 293T cells infected with a lentiviral vector encoding EGFP miR-T/mCherry miR-T were super-infected by a lentiviral vector encoding either *miR-302a*, *miR-302b*, *miR-302c*, and *miR-302d* (miR-302 a-d) or *miR-155* 2 days post-infection. Cells were then further cultured for 4 days and analyzed for EGFP and mCherry expression by flow cytometry. The number (%) in each quadrant is listed on each plot.

**Figure 2:**
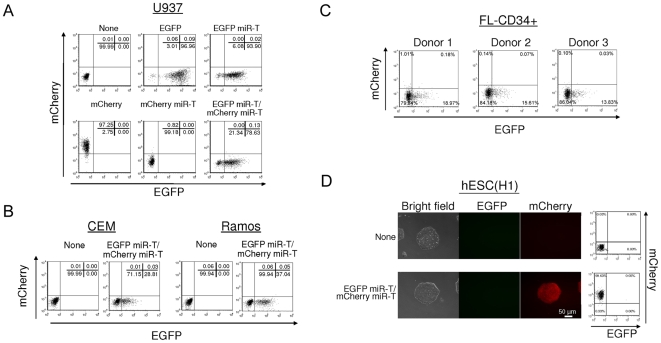
The reporter vectors containing miRNA targets show the lineage-specific expression. (A) U937 cells were infected with lentiviral vectors encoding various reporters showed in Fig. 1 A. Expression levels of EGFP and mCherry were analyzed by flow cytometry 2 days post-infection. (B and C) CEM and Ramos cells (B) and CD34+ HPSCs derived from 3 independent donors (C) were infected with a lentiviral vector encoding EGFP miR-T/mCherry miR-T. Expression levels of EGFP and mCherry were analyzed by flow cytometry 2 days post-infection. (D) hESCs (H1) were infected with a lentiviral vector encoding EGFP miR-T/mCherry miR-T. Single cell clone was isolated by culturing transduced cells in the presence of 10 µM Y27632 for 14 days. Expression levels of EGFP and mCherry were analyzed by fluorescence microscopy and by flow cytometry. The number (%) in each quadrant is listed on each plot.

We first demonstrated that this reporter construct is responsive to the endogenous miRNAs as predicted (Fig. 1). Both EGFP and mCherry are detected in 293T cells which express the relevant miRNAs at very low levels [18] (Fig. 1B). Ectopic expression of the miRNAs by co-transduction, either *miR-302a, miR-302b, miR-302c,* and *miR-302d* or *miR-155* results in ablation of EGFP or mCherry expression, respectively, demonstrating sensitivity of the vector to specific miRNAs (Fig. 1B, *miR-302a-d* and *miR-155*, respectively). In hematopoietic lineage cells (U937, monocyte lymphoma cell line) mCherry expression is entirely ablated whereas EGFP is maintained (Fig. 2A, mCherry miR-T and EGFP miR-T/mCherry miR-T). Similar results are seen in Ramos (B-cell lymphoma cell line) and CEM (T-cell lymphoma cell line) (Fig. 2B, EGFP miR-T/mCherry miR-T). We further tested the expression of EGFP miR-T/mCherry miR-T vector in CD34+ hematopoietic progenitor/stem cells (HPSCs) isolated from the fetal liver (FL-CD34+) (Fig. 2C). As expected, mCherry expression was strongly diminished, whereas EGFP was detectable in CD34+ HPSCs. In contrast, following transduction of hESC (H1 cell line), EGFP expression is fully ablated, whereas mCherry expression is maintained (Fig. 2D). We confirmed the phenotype by isolating and propagating the colonies and subjecting the cells to analysis by flow cytometry. The cells were maintained mCherry positive and EGFP negative for over 20 generations without notable adverse effects on their growth. Therefore, this reporter construct is suitable for assaying differentiation of hESC into particular lineages in a quantitative fashion utilizing a relatively small number of cells.

### miRNA dependent reporter expression during reprogramming of human fetal fibroblast (HFF) to hiPSC

**Figure 3:**
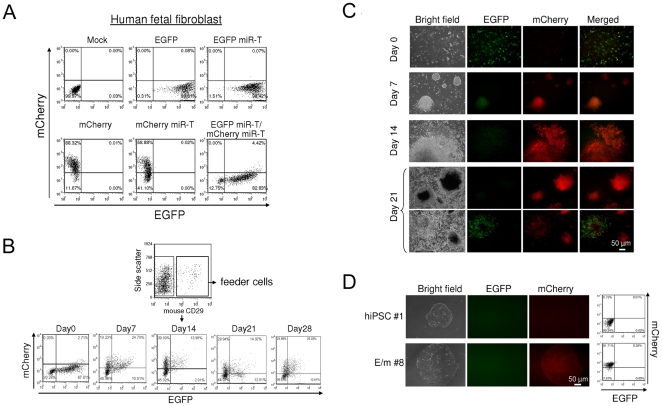
Reprogramming state specific expression of the reporter vector containing miRNA targets during hiPSC formation from human fetal fibroblasts (HFFs). (A) HFFs were infected with lentiviral vectors encoding various reporters shown in Fig. 1A. Expression levels of EGFP and mCherry were analyzed by flow cytometry 2 days post-infection. (B and C) HFFs were infected with lentiviral vectors encoding 4 different hiPSC factors (*OCT4, SOX2, KLF4,* and *cMYC*) and with or without a lentiviral vector encoding EGFP miR-T/mCherry miR-T. Cells were then cultured for 3 days and replated on irradiated mouse embryonic fibroblast (iMEF) feeder cells at 5×10^4^ cells/60 mm plate. Expression levels of EGFP and mCherry were analyzed by flow cytometry on days 0, 7, 14, 21, and 28 (B), and by fluorescence microscopy on days 0, 7, 14, and 21 (C). iMEF feeder cells were labeled with PE-Cy7 conjugated mouse CD29 antibody and excluded from the flow cytometry analysis in (B). (D) hiPSC colonies expressing mCherry were picked on Matrigel coated plate on days 21–25 and propagated in mTeSR medium. Expression levels of EGFP and mCherry were analyzed by fluorescence microscopy and by flow cytometry. The number (%) in each quadrant is listed on each plot. hiPSC #1: hiPSC clone without transduction of EGFP miR-T/mCherry miR-T reporter vector. E/m#8: hiPSC clone with transduction of EGFP miR-T/mCherry miR-T reporter vector.

The reprogramming of HFFs to hiPSCs can be achieved by the introduction of four transcription factors (*OCT4, SOX2, KLF4* and *cMYC*). After a prolonged period of culture on feeder cells, a fraction (<0.01%) of the cells reprograms and appears as hiPSC colonies [3], [6]. These hiPSCs have the characteristics of hESCs, including prolonged growth in culture and differentiation to multiple tissue lineages. We tested the ability of the reporter vector to distinguish between the terminally differentiated fibroblast starting population and hiPSC throughout the course of reprogramming (Fig. 3).

We first transduced HFFs derived from dermal skin with the reporter construct to assess its characteristics (Fig. 3A). Since the fibroblasts do not express *miR-302* [19], [20], [21], the cells were positive for EGFP (Fig. 3A, EGFP miR-T and EGFP miR-T/mCherry miR-T). We observed some ablation of mCherry expression due to low level expression of *miR-155* in fibroblasts [21] (Fig. 3A mCherry miR-T and EGFP miR-T/mCherry miR-T). EGFP expression was robust and would be predicted to be extinguished during reprogramming to hiPSC.

We transduced HFFs with the reporter vector concomitantly with vectors expressing the four hiPSC reprogramming factors *(OCT4, SOX2, KLF4*, and *cMYC*). Expression levels of EGFP and mCherry were monitored over the four week course of reprogramming to assess the activity of the reporter vector during generation of hiPSC (Figs.3B and 3C). We observed a decrease in EGFP expression over time, presumably reflecting the induction of the hESC specific miRNAs, including *miR-302a/d*. In addition, mCherry was partially extinguished in the HFF (Fig. 3B, Day0) and over time we observed an increase in mCherry expression (Fig. 3B, Days 7, 14, 21, and 28), presumably reflecting loss of *miR-155* which is expressed only in the latest stages of differentiation and not in cells with characteristics of pluripotent stem cells [21].

Interestingly, our results show that many cells expressed the hESC-specific *miR-302a/d* as evidenced by reduction of EGFP expression from day 0 to day 21 (70% to 27%, Fig. 3B), but only a small fraction of those cells actually formed hESC-like colonies. In addition to these colonies, a greater number of colonies of transformed phenotype, characterized by large granulated colonies, were observed, similar to a previous report [3]. Among these colonies, approximately 90% were EGFP negative and mCherry positive, indicating expression of *miR-302a/d* and no expression of the differentiation specific miRNAs, suggesting a partially reprogrammed state for these transformed cells as previously reported [19], [28], [29]. A fewer number of transformed colonies were positive for both EGFP and mCherry (see example Fig. 3C, left side colony in bottom panels of Day21). Colonies with the distinctive morphologic appearance of hiPSC, characterized by small and tightly packed colonies with smooth borders, were isolated from the culture on day 21–25. The frequency of hiPSC formation induced by four factors plus the reporter vector was approximately 0.03%. Nearly 100% of the morphologically distinct hiPSC colonies were EGFP negative and mCherry positive (Fig. 3D). We isolated and propagated 13 hiPSC clones with transduction of EGFP miR-T/mCherry miR-T lentiviral vector and 20 hiPSC clones without transduction of the reporter vector.

**Figure 4:**
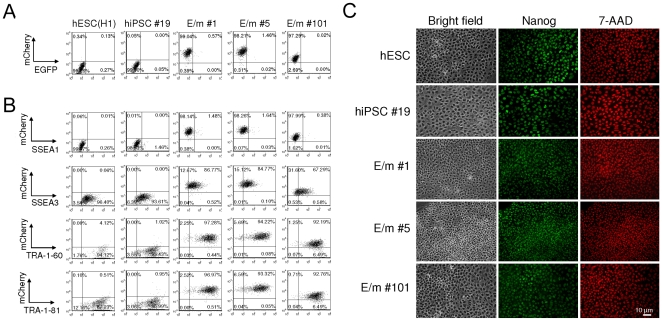
Transduction of the reporter vector containing miRNA targets does not grossly affect expression of hESC-specific markers. (A and B) Single-cell suspensions of hESC (H1), hiPSCs transduced with a lentiviral vector encoding EGFP miR-T/mCherry miR-T (E/m#1, E/m#5, and E/m#101) or untransduced (hiPSC#19) were analyzed for the expression of EGFP and mCherry (A) and that of hESC-specific markers (SSEA1, SSEA3, TRA1-60, and TRA-1-81) (B) by flow cytometry. The number (%) in each quadrant is listed on each plot. (C) hESCs (H1), hiPSCs transduced with a lentiviral vector encoding EGFP miR-T/mCherry miR-T (E/m#1, E/m#5, and E/m#101) or untransduced (hiPSC#19) were plated on poly-L-lysine and Matrigel coated glass coverslips and expanded for a week. Cells were then fixed with 1% formaldehyde, permeabilized with 0.2% Triton X-100 for 5 min on ice, and stained with anti-Nanog antibody and DyLight488 conjugated anti-rabbit IgGs. 7-amino-actinomycin D (7-AAD) was used for nuclear staining.

**Figure 5:**
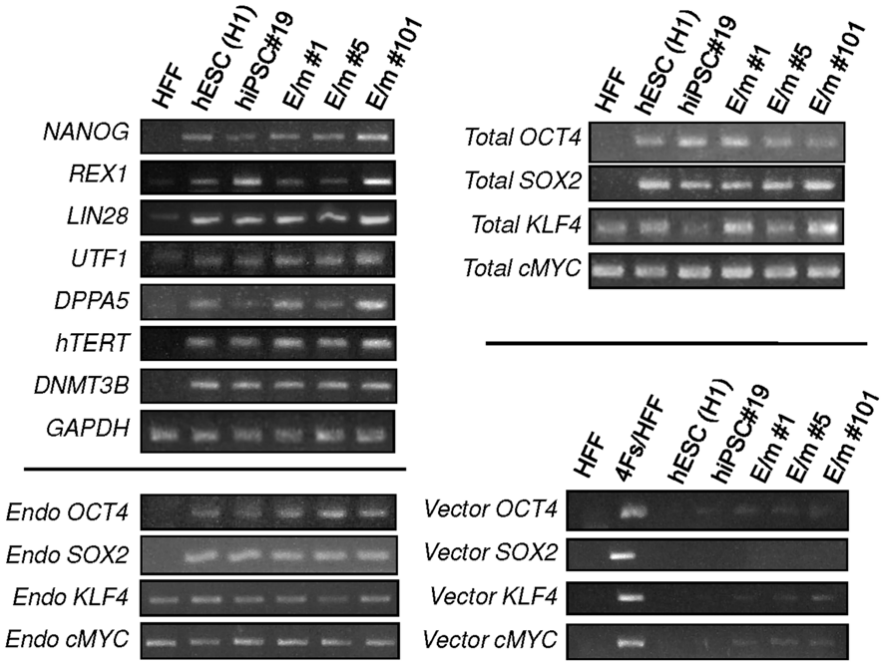
Molecular characterization of hiPSCs transduced with the reporter vector containing miRNA targets. Total RNA was isolated using QIAGEN's RNeasy Mini kit from HFFs transduced with (4Fs/HFF) or without 4 reprogramming factors (HFF), hESCs (H1), and 4 different hiPSC clones transduced with (E/m#1, E/m#5, and E/m#101) or without (hiPSC#19) the reporter vector encoding EGFP miR-T/mCherry miR-T. Total RNA (250 ng) was reverse-transcribed using QIAGEN's Omniscript reverse transcription kit and used as a template in subsequent PCR with 5-PRIME's HotMaster Taq DNA polymerase. PCR products were analyzed on a 2% agarose gel. Glyceraldehyde-3-phosphate dehydrogenase (GAPDH) was used as an internal control.

We analyzed three out of 13 hiPSC clones transduced with the reporter vector (E/m #1, E/m #5, and E/m #101) and one hiPSC clone that was not transduced with this reporter vector (hiPSC #19) for characteristics of pluripotent stem cells. These hiPSC clones transduced with the reporter vector stably maintained mCherry expression and lack of EGFP expression for over 20 generations (Fig. 4A). Although all the clones were EGFP negative, there were some differences in the expression levels of mCherry. For example, E/m #101 clone had reduced mCherry expression compared to other iPSC clones (Fig. 4A). However, by cell surface staining, they were almost all negative for SSEA1 and mostly positive for SSEA3, TRA-1-60, and TRA-1-81 consistent with the phenotype of hESC (Fig. 4B). Furthermore, all clones including the remaining 10 clones transduced with the reporter vector and 19 clones without transduction of the reporter vector (data not shown), were Nanog positive confirmed by indirect immunofluorescent staining (Fig. 4C). Reverse transcriptase PCR (RT-PCR) analysis of these clones confirmed expression of hESC-specific mRNAs (Fig. 5). The clones were positive for *NANOG, REX1, LIN28, UTF1, DPPA5, hTERT, DNMT3B, OCT4* and *SOX2* similar to the control hESC H1 line. In contrast, HFFs were positive for *KLF4* and *cMYC* as reported [1], [30]. These results indicate that ectopic expression and the expression levels of this reporter vector do not grossly affect either hiPSC induction or the expression of hESC-specific markers.

### The reporter vector indicates differentiation of hiPSC into EBs and into neural lineages

**Figure 6:**
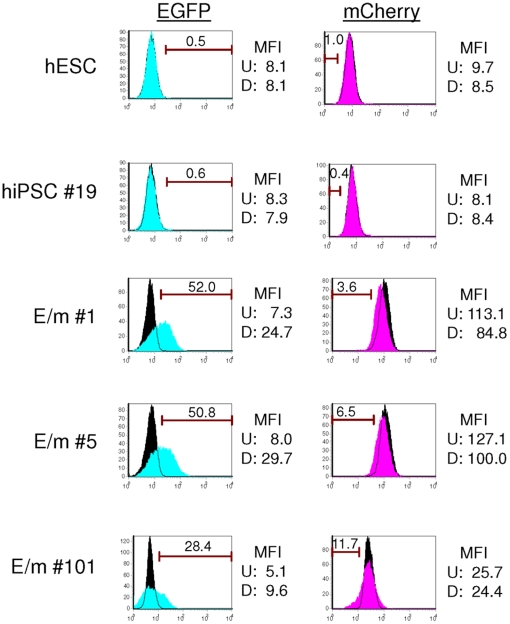
hiPSCs transduced with the reporter vector containing miRNA targets show differentiation-specific reporter expression in EBs. hESC (H1) and 4 different hiPSC clones (E/m#1, E/m#5, E/m#101, and hiPSC#19) were differentiated into EBs and maintained 25 days in IMDM containing 10% FBS. EBs were then dissociated with 0.25% trypsin/EDTA and the reporter expression was analyzed by flow cytometry. Histograms filled with black are undifferentiated controls. Histograms filled with blue (EGFP) and pink (mCherry) are differentiated cells, respectively. The numbers indicated in histogram show percentage of positive cells (EGFP) and negative cells (mCherry). MFI: mean fluorescence intensity. U: MFI of undifferentiated cells. D: MFI of differentiated cells.

hESC as well as hiPSC can be differentiated *in vitro* into EBs comprising the three embryonic germ layers [3], [31]. We first tested whether the hiPSC clones transduced with the reporter vector can be differentiated into EBs and whether expression of the reporter vector is dependent upon the miRNA expression profile (Fig. 6). Our results show that upon differentiation of the hiPSC into EBs for 25 days, the majority of the cells harboring the reporter vector now expressed EGFP, presumably reflecting the loss of *miR-302a/d* expression as the cells differentiated. Concomitantly, mCherry expression was slightly reduced in the cells, reflecting expression of one or more of the differentiation specific miRNAs *miR-223, miR-155*, or *miR-142-3p*. However, since the EBs represent multiple lineages of differentiated cells, we were unable to conclude which cells and to what extent these miRNAs are being expressed.

**Figure 7:**
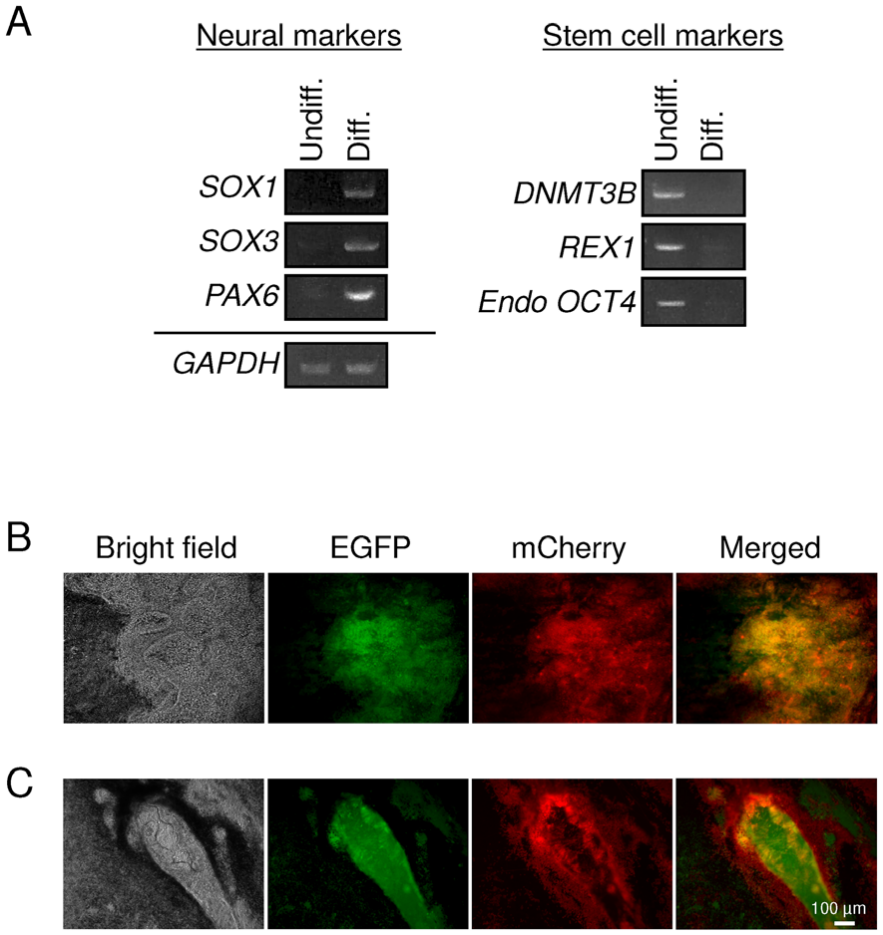
hiPSCs transduced with the reporter vector containing miRNA targets indicate differentiation-specific reporter expression in neural lineages. EBs generated from pooled hiPSCs were differentiated into neural lineages using Noggin and SB431542. EBs were then transferred onto fibronectin coated 6-well plates and further differentiated in N2 medium. (A) Total RNA was isolated using QIAGEN's RNeasy Mini kit from the hiPSCs on day 0 (undifferentiated population: Undiff.) and day 30 (differentiated population: Diff.) after induction of differentiation. Total RNA (250 ng) was reverse-transcribed using QIAGEN's Omniscript reverse transcription kit and used as a template in subsequent PCR with 5-PRIME's HotMaster Taq DNA polymerase. PCR products were analyzed on a 2% agarose gel. GAPDH was used as an internal control. (B) Neural tube-like rosettes observed after the differentiation. (C) Dark pigmented melanocyte-like cells surrounding neural tube-like structures.

We further tested the reporter expression in neural lineages. Differentiation into neural lineages from hESCs and hiPSCs can be induced by culturing them in the presence of Noggin and transforming growth factor-β inhibitor, SB431542, both of which are inhibitors of SMAD signaling [32]. Under this condition, hESCs and hiPSCs organize into neural tube-like rosettes identified as neural progenitor cells [33], [34], [35]. With further differentiation, neural rosettes produce neural crest-progenitor cells which give rise to diverse derivatives, such as the peripheral nervous system, melanocytes, and cranial mesenchymal cells [36], [37], [38], [39]. We generated EBs from hiPSCs transduced with the reporter vector and induced differentiation into neural lineages by culturing them in the presence of Noggin and SB431542. The differentiation status of the EBs into neural lineages was monitored by the expression of *SOX1*, *SOX3* and *PAX6*, markers of neuroectodermal differentiation [40], whereas the undifferentiated iPSCs were monitored by the expression of *DNMT3B, REX1* and endogenous *OCT4* which are assumed to be expressed in fully-reprogrammed hiPSCs [41]. One month after induction of differentiation, *DNMT3B*, *REX1* and endogenous *OCT4* were downregulated, whereas *SOX1*, *SOX3* and *PAX6* were upregulated in the differentiated population compared to the undifferentiated population (Fig. 7A). Neural tube-like rosettes were observed throughout the culture plate, most of which were both EGFP and mCherry positive (Fig. 7B), indicating that they do not express any miRNA that recognizes the targets in the EGFP miR-T/mCherry miR-T reporter vector. We observed dark pigmented melanocyte-like cells surrounding neural tube-like structures as previously reported [32] (Fig. 7C). Interestingly, the fluorescence of EGFP and mCherry allowed a clear demarcation of boundaries between apparently different structures within the neural tube-like structure and the surrounding dark pigmented area. The cells located around the edge of the neural tube-like structure were mostly double positive for EGFP and mCherry. In contrast, the cells located at the inner side of the neural tube-like structure were EGFP-single positive. The majority of melanocyte-like cells surrounding neural tube-like structures were mCherry-single positive. Expression of the specific miRNAs tested here is unknown within the neural tube-like structures, however there appears to be expression of one or more differentiation specific miRNAs in the interior whereas *miR-302a/d* appears to be expressed in the surrounding dark pigmented area. Therefore, these results demonstrate the use of the reporter vector to provide real-time observation of hiPSC differentiation.

## Discussion

Our results demonstrate the utility of a miRNA dependent reporter vector system to monitor reprogramming of human fibroblasts to hiPSC and subsequent differentiation of the hiPSC into multi-lineage embryoid bodies. Loss of EGFP expression that is dependent upon expression of the hESC-specific *miR-302a/d*, is highly specific to the pluripotent cells. There is robust EGFP expression observed in HFFs and significant levels in differentiated EBs and neural-lineage cells, but no detectable expression in hESC or the hiPSC. In contrast, the hiPSCs express mCherry at high levels similar to hESCs, reflecting the absence of *miR-223*, *miR-155*, or *miR-142-3p* in the hiPSCs. Differentiated cells, including HFF and some cells in EBs express some of these miRNAs, resulting in downregulation of mCherry expression.

The miRNA dependent reporter vector can be used to study stages of reprogramming and differentiation since it allows for assay and/or isolation of cells based upon fluorescence intensity. It is noteworthy that the hESC-specific miRNAs, *miR-302a/d*, are expressed in a significant proportion of the cells during the process of reprogramming. Consistent with previous studies [19], [42], these results indicate that expression of hESC-specific factors, including miRNAs, occurs in the majority of cells as a result of the ectopic introduction of reprogramming factors, but a much smaller percentage of those cells reprogram properly to form hiPSC. In our studies, more than 50% of the cells express *miR-302a/d* based upon loss of EGFP during reprogramming at day 14, but only 0.03% of the starting HFF result in hiPSC. These results indicated that *miR-302a/d* is not sufficient for reprogramming and therefore cannot be used solely as a reporter to identify true hiPSC. Future more selective choice of miRNAs in combination with *miR-302 a/d* may be utilized to fractionate hiPSC from partially reprogrammed cells based upon their expression profile of fluorescence and to further investigate reprogramming mechanisms. Similarly, differentiated cells can be fractionated for further investigation based upon the lineage restricted expression of specific miRNAs.

Ectopic expression of miRNA target sequences may be of concern in potentially interfering with the endogenous miRNA machinery. Other investigators have not noted alterations in cellular miRNA metabolism with relatively high copy number [17]. Nevertheless, the use of vectors and coding miRNA targets should ideally be utilized at low copy number per cell and should be monitored for any effects upon cellular differentiation and/or function. In our case, all hiPSC clones transduced with the reporter vector were well-maintained under hESC culture conditions similar to non-transduced hESC or hiPSC. These hiPSCs expressed multiple hESC-specific mRNAs and antigens, like Nanog, SSEA3, SSEA4, TRA-1-60 and TRA-1-81. Furthermore, these cells were able to differentiate into neural cells and beating cardiomyocytes (data not shown), identically to control cells, indicating that the expression of miRNA targets did not grossly affect their differentiation abilities.

Interestingly, one of the hiPSC clones (E/m #101) had reduced mCherry expression compared to two other hiPSC clones transduced with the reporter vector (Fig. 4A). Although this clone resembled the other hiPSC clones and hESCs in regards to expression of hESC-specific markers, SSEA3 expression was slightly lower than that of other clones or hESCs (Fig. 4B). Furthermore, this clone had some morphological differences - the colonies of this clone were flatter compared to those of hESCs or hiPSCs and the boundaries between cells which are indistinguishable for hESCs and other hiPSCs all over the colonies were distinct especially at the outer edge of the colonies (data not shown). Growth and propagation of this clone also resulted in a greater level of spontaneous differentiation than that of the other clones. Moreover, this clone showed a weaker shift of EGFP and mCherry expression upon differentiation into EBs (Fig. 6). These results suggest that functional assays based upon endogenous miRNA expression may be another means to assess properties of hiPSC.

The development of this vector adds to the tools available to monitor hiPSC generation and subsequent differentiation of the hiPSC into different lineages. In addition to taking advantage of differential miRNA expression, other investigators have utilized differential promoters/enhancer expression in different lineages [43], [44]. Whether one utilizes miRNA or promoter/enhancer expression of reporter constructs for live-cell tracking of reprogramming and differentiation will depend on the particular experimental setting and application.

The work presented here indicates the potential great utility and flexibility of miRNA-regulatable lentiviral vectors to monitor various stages of reprogramming and the subsequent differentiation into lineage specific cells and tissues. For example, *miR-223, miR-142-3p,* and *miR-155* are enriched primarily in cells of hematopoietic origin [22], [23]. The expression of mCherry conjugated with these miR targets was strongly suppressed in CD34+ HPSCs (Fig. 2 C). These results suggest that our reporter vector can be used to monitor the differentiation into CD34+ HPSCs from hESCs/hiPSCs, and to isolate low frequency populations of cells based upon the differential expression of EGFP and mCherry. Further selective use of miRNA targets would be predicted to preferentially suppress expression in specific lineages or specific stages of differentiation within a given lineage. Thus, the activity of the reporter vector can be readily modulated depending upon the miRNA target sequence incorporated and such a vector can be used for studies involving specific differentiation lineages where the miRNA expression profile is known. Conversely, a vector with a specific target sequence can be used to determine the temporal and lineage specific expression of the corresponding miRNAs during differentiation.

## Materials and Methods

### Construction of lentiviral vector

For construction of the reporter vector, whole sequences of hESC specific-miRNA targets (CCCGGGCGAGCAAGCTCAGTTTACACCGAATTCGGATCCTCACCAAAACATGGAAGCACTTAAGTCTCACCAAAACATGGAAGCACTTAAGTCTCACCAAAACATGGAAGCACTTAAGTCTCACCAAAACATGGAAGCACTTAGGCCTACACTCAAACATGGAAGCACTTAGTACACACTCAAACATGGAAGCACTTAGTACACACTCAAACATGGAAGCACTTAGTACACACTCAAACATGGAAGCACTTAGATATCGTCGAC) and differentiated cell specific-miRNA targets (CCCGGGTCGAATTCGGTACCAGATCTGGCGCGCCGTACGTGGGGTATTTGACAAACTGACAAGTCTGGGGTATTTGACAAACTGACAAGTCTGGGGTATTTGACAAACTGACAAGTCTGGGGTATTTGACAAACTGACAGGCCTACCCCTATCACGATTAGCATTAAAGTCACCCCTATCACGATTAGCATTAAAGTCACCCCTATCACGATTAGCATTAAAGTCACCCCTATCACGATTAGCATTAATTTAAATTCCATAAAGTAGGAAACACTACAGTACTCCATAAAGTAGGAAACACTACAGTACAGTTCCATAAAGTAGGAAACACTACAGTACTCCATAAAGTAGGAAACACTACAGATATCTGCATGCTTCGAAGCTAGCGGGCCC) were synthesized by Genescript (Piscataway, NJ). The synthesized fragment of differentiated cell specific-miRNA targets was conjugated to mCherry coding sequence amplified by PCR from pmCherry (Clontech Laboratories, Inc. Mountain View, CA) with primers (sense: ACGCACCGGTGGATCCAAGCTTGCCACCATGGTGAGCAAGGGCGAGGA and reverse: CTGCGAATTCTCACTACTTGTACAGCTCGTCCATGCCGCCGG) and exchanged with EGFP coding sequence in FG12 lentiviral vector [45] (mCherry-T/FG12). For bidirectional expression from one promoter [46], EGFP coding sequence was amplified by PCR from the pEGFP-N1 (Clontech Laboratories) with primers (sense: AGTCAGCTAGCGCCACCATGGTGAGCAAGGGCGAG and reverse: CATCGACCCGGGAATTCTCATTACTTGTACAGCTCGTCCATG), and cloned into a pAAV-MCS vector (Stratagene, La Jolla, CA) (pAAV-EGFP). The synthesized fragment of hESC specific-miRNA targets was inserted into the pAAV-EGFP between stop codon of EGFP and hGH poly A signal. The fragment containing CMV minimal promoter, β-globin intron, EGFP, hESC specific-miRNA targets, and hGH poly A signal was amplified by PCR with primers (sense: GTACTCTCGAGCCCCATTGACGCAAATGGGCGGTAGG and reverse: CTCGTCTAGAAGGACAGGGAAGGGAGCAGTGGT) and cloned into the FG12 lentiviral vector deleted EGFP (EGFP-T/FG12) or into the mCherry-T/FG12 (EGFP-T/mCherry-T/FG12).

For reprogramming of HFFs, we substituted the ubiqutin C promoter of FG12 lentiviral vector with the RhMLV promoter (FRh11). The RhMLV promoter is derived from the long terminal repeat (LTR) region of Moloney murine leukemia virus (MLV) in the serum of one rhesus macaque monkey that developed T-cell lymphoma following autologous transplantation [47], [48]. This promoter shows around 5–10 fold stronger promoter activity in HFFs compared to that of the parental MLV LTR (unpublished observation). Furthermore, this promoter activity is strongly silenced in hESC or hiPSC as well as that of the parental MLV LTR (unpublished observation). cDNAs encoding human *OCT4*, *SOX2*, *KLF4*, and *cMYC* (Addgene) were substituted with EGFP coding sequence in the FRh11. The infectious titer was determined in 293T cells by infecting with the FRh11 encoding EGFP in the presence of 8 µg/ml polybrene. Reporter gene expression was monitored by flow cytometry.

For ectopic expression of miRNAs, we purchased miRNA expressing lentiviral vectors for *miR-302a, miR-302b, miR-302c,* and *miR302-d* (PMIRH302abcdPA-2) and *miR-155* (PMIRH155PA-1) from System biosciences (Mountain View, CA). CopGFP sequence was eliminated from the vector.

### Cell culture

293T [49], Ramos [50], U937 [51] and CEM [52] cells were maintained with Dulbecco's Modified Eagle Medium (DMEM) (Invitrogen, Carlsbad, CA) supplemented with 10% fetal bovine serum (FBS) (Omega Scientific, Tarzana, CA) and 2 mM GlutaMax (Invitrogen). All cells were incubated at 37°C in 5% CO_2_.

hESCs (H1 clone) [53] and hiPSCs were maintained in mTeSR™1 (StemCell Technologies, Inc., Vancouver, Canada) on hESC-qualified Matrigel™ (BD Biosciences, San Jose, CA) coated plates. Differentiated colonies were removed daily through aspiration, and the medium was replaced on a daily basis. Cells were passed upon confluency (typically 7–10 days), using 1 mg/ml dispase (StemCell Technologies, Inc.). All work with hESC and hiPSC was approved by the UCLA Embryonic Stem Cell Research Oversight committee.

HFFs were isolated from the skin of 16 week-old fetus with DMEM supplemented10% FBS and 2 mM Glutamax (fibroblast medium) as reported previously [54].

CD34+ HPSCs were prepared from the liver of 16 weeks-old fetus as previously described [55].

### Virus production

Lentiviral vector stocks were generated using a vector plasmid, a packaging plasmid pCMV R8.2 ΔVpr, and a VSV-G envelope protein-coding plasmid by calcium phosphate-mediated transient transfection as previously described [56]. After 48 and 72 hr, lentiviral vector particles were harvested and concentrated by ultracentrifugation and resuspended in a 150-fold lower volume of Hanks' balanced salt solutions and stored at −80°C. The viral titer was measured by anti-p24 Gag ELISA.

### Induction of hiPSC

The day before lentiviral vector transduction, HFFs (passage 1–3) were seeded at 5×10^4^ cells per well of 6-well plates and infected with vectors encoding each reprogramming factor (*OCT4, SOX2, KLF4,* and *cMYC*) with or without a lentiviral vector encoding EGFP-T/mCherry-T at 300 ng (around multiplicity of infection of 3–5) of p24 per each virus. The cells were cultured for 3 days in fibroblast medium and replated at 5×10^4^ cells per 60 mm dish on irradiated mouse embryonic fibroblast (iMEF) feeder cells. On the next day, the medium was replaced with KO-DMEM (Invitrogen) supplemented with 20% Knockout Serum Replacer (KSR, Invitrogen), 2 mM Glutamax (Invitrogen), 0.1 mM non-essential amino acids (Invitrogen), 0.1 mM β-mercaptoethanol (Sigma-Aldrich, St. Louis, MO), and 50 ng/ml of recombinant human basic fibroblast growth factor (Invitrogen) (hiPSC medium). The medium was changed on a daily basis. To increase a reprogramming efficiency, the cells were treated with 0.5 mM valproic acid (VPA; Sigma-Aldrich) and 10 µM Y27632 (Tocris Bioscience, Ellisville, MO) for first 14 days [57], [58]. On day 21–25, hiPSC colonies were identified based upon hESC-like morphology as described previously [3] and picked out into wells of 48-well plates coated with Matrigel and expanded in mTeSR medium. Reprogramming efficiency was calculated as the number of hiPSC colonies formed per number of seeded HFFs with transduction of reprogramming factors.

### Embryoid body formation

Size-controlled EBs (3000 hESCs/EB) were formed using AggreWell™ 400 plates (StemCell Technologies, Inc.) following the manufacturer's protocol. Briefly, hESCs and hiPSCs were incubated with 10 µM Y-27632 for 24 hrs before EB formation. Cells were harvested with Accutase (Innovative Cell Technologies, San Diego, CA) as a single-cell suspension and used for EB formation. EBs were harvested into ultra low attachment plates (Corning, Corning, NY) and maintained in Iscove's Modified Dulbecco's Medium (IMDM, Sigma-Aldrich) containing 10% FBS, 2 mM Glutamax, and 0.1 mM β-mercaptoethanol for differentiation into EBs. The medium was changed every 3 days.

### Differentiation into neural lineages

Induction of differentiation into neural lineages was performed as previously described with some modifications [32]. Briefly, EBs were maintained in the hiPSC medium containing 500 ng/ml of Noggin (R&D) and 10 µM SB431542 in ultra low attachment plates. SB432542 was withdrawn on day 5 and increasing amounts of N2 medium (Stem cell technologies) (25%, 50%, 75%) was added to the hiPSC medium every 2 days while maintaining 500 ng/ml of Noggin. Upon day 12 of differentiation, EBs were transferred onto fibronectin-coated 6-well plates and maintained in 100% N2 medium without Noggin for further 18 days.

### Flow Cytometry

For detection of EGFP and mCherry expression, single-cell suspensions from 293T, HFF, hESC, and hiPSC were prepared using 0.25% trypsin-EDTA and collected in FACS buffer (2% FBS and 0.01% sodium azide in PBS). U937, Ramos and CEM cells were also collected in FACS buffer. For detection of hESC-specific markers, hESCs and hiPSCs were dissociated with 0.25% trypsin-EDTA into a single cell suspension. Cells were adjusted to 100,000 per sample in 100 µl of FACS buffer and then labeled with monoclonal antibodies conjugated with a fluorescent dye [SSEA1 and SSEA3: Alexa488 purchased from eBioscience (San Diego, CA); TRA-1-60 and TRA-1-81: PE purchased from BioLegend (San Diego, CA)]. For detection of iMEFs, cells were stained with an antibody specific with mouse CD29 conjugated with PE-Cy7 (eBioscience). Data were collected on a Cytomics FC500 (Beckman Coulter, Fullerton, CA) and analyzed using FCS express (De Novo Software, Los Angeles, CA).

### Immunocytochemistry

hESC and hiPSC colonies were grown on poly-L-lysine and Matrigel coated glass coverslips. Cells were fixed with 1.0% formaldehyde/PBS and permeabilized with 0.2% Triton-X 100 for 5 min on ice. Cells were then incubated with anti-human Nanog antibody (Abcam Inc., Cambridge, MA) and subsequently with DyLight488 conjugated donkey anti-rabbit IgG (BioLegend) and 7-amino-actinomycin D (7-AAD) (Invitrogen) for nuclear staining. After washing, cells were visualized with a LEICA DM IRB (Leica Microsystems Inc. Bannockburn, IL) equipped with a SPOT camera and software (Diagnostic Instruments, Sterling Heights, MI).

### RNA extraction and RT-PCR

RNA extraction from hESC and hiPSC was performed using QIAGEN's RNeasy Mini kit following the manufacture's protocol (QIAGEN, Valencia, CA). Total RNA (250 ng) was reverse-transcribed using QIAGEN's Omniscript RT-kit with a 0.5 ng/ml oligo dT primer (Invitrogen) in 20 µl reaction. PCR was performed with the HotMaster Taq DNA polymerase (5 PRIME, Inc., Gaithersburg, MD), using 0.5 µl of cDNA template and primers at a concentration of 3 pmol/µl. Five µl of PCR products was loaded in a 2% agarose gel containing ethidium bromide. All primer sequences were listed in Table 1.

**Table 1:** Primers for RT-PCR.

Genes	Forward (5′ to 3′)	Reverse (5′ to 3′)	Size (bp)
Endogenous			
*NANOG*	CAGCCCTGATTCTTCCACCAGTCCC	GGAAGGTTCCCAGTCGGGTTCACC	390
*REX1*	CAGATCCTAAACAGCTCGCAGAAT	GCGTACGCAAATTAAAGTCCAGA	305
*LIN28*	ATCTGTAAGTGGTTCAACGTGCGC	TGGCAGCTTGCATTCCTTGGCATG	338
*UTF1*	CCGTCGCTGAACACCGCCCTGCTG	CGCGCTGCCCAGAATGAAGCCCAC	147
*DPPA5*	ATATCCCGCCGTGGGTGAAAGTTC	ACTCAGCCATGGACTGGAGCATCC	242
*hTERT*	TGTGCACCAACATCTACAAG	GCGTTCTTGGCTTTCAGGAT	165
*DNMT3B*	ATAAGTCGAAGGTGCGTCGT	GGCAACATCTGAAGCCATTT	121
*OCT4*	CCTCACTTCACTGCACTGTA	CAGGTTTTCTTTCCCTAGCT	163
*SOX2*	CCCAGCAGACTTCACATGT	CCTCCCATTTCCCTCGTTTT	150
*cMYC*	TGCCTCAAATTGGACTTTGG	GATTGAAATTCTGTGTAACTGC	191
*KLF4*	GATGAACTGACCAGGCACTA	GTGGGTCATATCCACTGTCT	144
*GAPDH*	GAAGGTGAAGGTCGGAGT	GAAGATGGTGATGGGATTTCC	225
Total			
*OCT4*	AGCGAACCAGTATCGAGAAC	TTACAGAACCACACTCGGAC	140
*SOX2*	AGCTACAGCATGATGCAGGA	GGTCATGGAGTTGTACTGCA	125
*cMYC*	ACTCTGAGGAGGAACAAGAA	TGGAGACGTGGCACCTCTT	158
*KLF4*	TCTCAAGGCACACCTGCGAA	TAGTGCCTGGTCAGTTCATC	104
Vector			
*OCT4*	GCTCTCCCATGCATTCAAACTGAGG	GGAGCAACATAGTTAAGAATACCAGTC	167
*SOX2*	GACTTCACATGTCCCAGCACTACC	GGAGCAACATAGTTAAGAATACCAGTC	361
*cMYC*	GAACAGCTACGGAACTCTTGTGCG	GGAGCAACATAGTTAAGAATACCAGTC	203
*KLF4*	AGCATTTTCCAGGTCGGACCACC	GGAGCAACATAGTTAAGAATACCAGTC	328
Neural lineage			
*SOX1*	CAATGCGGGGAGGAGAAGTC	CTCCTCTGGACCAAACTGTG	466
*SOX3*	ACCTTTGTAGGCTGGGAATCG	ATCACGGCAGAAATCACCAAC	365
*PAX6*	GCCAGCAACACACCTAGTCA	TGTGAGGGCTGTGTCTGTTC	136
